# Supplementary material for: A multi-country qualitative evaluation of rapid mortality surveillance during the COVID-19 pandemic
Source: PLoS One. 2026 Jul 6;21(7):e0333157. doi: 10.1371/journal.pone.0333157 (PMC13336173; doi:10.1371/journal.pone.0333157)
Supplement: Attachment — Submitted filename: Response to Reviewers.docx [file pone.0333157.s001.docx]

**Review Comments to the Author, with Response**

Submitted 3/3/2026

**Reviewer #1:** The article as a whole is more like a research report than an academic paper, and I am puzzled by the scientific nature of the methodology used in the article, and the relatively small amount of work that went into the article. Please enrich the article further by taking into account the comments of other reviewers, and I will give my next revision after the overall improvement.

**Response:** This was a both a summative evaluation and a utilization-focused process evaluation. It was a summative evaluation in that it looked back to systematically describe specific outcomes and test assumptions following Rapid Mortality Surveillance (RMS) implementation efforts during a global pandemic. It is utilization-focused process evaluation that assessed how RMS work was implemented and determined what components of RMS system design and implementation contributed to or hindered the production of timely, quality data that meets the goals of RMS. In that way, countries and global partners can use these results to inform future strategies and the refinement of global guidance on RMS.

As noted by Reviewer #3, the question about statistical analysis is N/A because this was a qualitative study with double-blind coding of in-depth interviews that focused on implementation methods.

**Reviewer #2:** 1. Correct typos/duplications: “low- and middle-income income countries”; “evidence-informed informed risk ratings.”

**Response:** This has been corrected.

2. Harmonize spelling/usage: choose digitization or digitalization and use consistently; standardize “COVID-19” (no space before/after hyphen).

**Response:** This has been corrected.

3. Add footnotes to define “Number of deaths recorded through 2022” (RMS-captured deaths vs. national totals; inclusion/exclusion of community deaths; duplication handling).

**Response:** This has been done.

4. Figure Suggestion - Add a simple schematic/logic model showing inputs → processes → outputs → outcomes for RMS (with facilitators/barriers around it). This helps generalizability beyond COVID-19

**Response:** This has been added to page 5

5. “COVID -19” → “COVID-19” (remove stray spaces).

**Response:** We have reviewed the document and confirmed there are no instances of stray spaces, “COVID-19” is consistently used.

6. Country names are listed however quotes are region-attributed, add one sentence explaining the anonymization rationale, and to confirm transcripts were de-identified and stored securely with restricted access.

**Response:** This has been done. The anonymization rationale is mentioned in a footnote to the first quote, and a sentence on data security has been added to the Methods section.

7. General Specificity Improvements may be prudent - namely with acronyms like first mention of CRVS, and throughout the table with footnotes

**Response:** We’ve ensured that each acronym is defined at first use. Additional details have also been added to Table headings, including footnotes where needed to define methodology or for added clarity.

8. Check punctuation around ellipses in quotations and bracket conventions.

**Response:** This has been done, we’ve ensured consistent spacing in each quotation.

9. Replace passive/wordy phrases where meaning can be tightened (e.g., “were able to be operationalized” → “were operationalized”).

**Response:** This point is well taken however in the example above the ability to be operationalized or not was an important distinction, as some countries tried to operationalize RMS systems but were unable. We’ve tightened the wording to “could” in this instance and reviewed for others.

10. Ensure consistent use of Em-Dashes vs. Hyphens

**Response:** This has been done.

**Reviewer #3:** The manuscript provides an extremely valuable and technically sound qualitative process review of the deployment of Rapid Mortality Surveillance (RMS) in 13 different nations. The approach, which included double-blind coding and in-depth interviews, was carried out carefully and is suitable for this kind of study. The conclusions, which suitably draw from the thematic findings, offer compelling proof of the crucial roles that government ownership, digitalization, and interagency cooperation have in the success of RMS.

The question about statistical analysis is N/A because this is a qualitative study that focuses on implementation methods.

The manuscript's important findings are accessible since it is written in clear, standard English and presented in an understandable manner.

**Response:** Thank you very much for the comprehensive and positive feedback, this is well noted.
