# Supplementary material for: A multi-country qualitative evaluation of rapid mortality surveillance during the COVID-19 pandemic
Source: PLoS One. 2026 Jul 6;21(7):e0333157. doi: 10.1371/journal.pone.0333157 (PMC13336173; doi:10.1371/journal.pone.0333157)
Supplement: Peer review history

# Peer review: Decision Letter 0

**DOI:** 10.1371/journal.pone.0333157.r001

**Authors:** Pasyodun Koralage Buddhika Mahesh

19 Jan 2026

PONE-D-25-49179

A Multi-Country Qualitative Evaluation of Rapid Mortality Surveillance During the COVID -19 Pandemic

PLOS One

Dear Dr. Congdon,

Thank you for submitting your manuscript to PLOS ONE. After careful consideration, we feel that it has merit but does not fully meet PLOS ONE’s publication criteria as it currently stands. Therefore, we invite you to submit a revised version of the manuscript that addresses the points raised during the review process.

Please submit your revised manuscript by Mar 05 2026 11:59PM. If you will need more time than this to complete your revisions, please reply to this message or contact the journal office at plosone@plos.org. When you're ready to submit your revision, log on to https://www.editorialmanager.com/pone/ and select the 'Submissions Needing Revision' folder to locate your manuscript file.

Please include the following items when submitting your revised manuscript:

- A letter that responds to each point raised by the academic editor and reviewer(s). You should upload this letter as a separate file labeled 'Response to Reviewers'.
- A marked-up copy of your manuscript that highlights changes made to the original version. You should upload this as a separate file labeled 'Revised Manuscript with Track Changes'.
- An unmarked version of your revised paper without tracked changes. You should upload this as a separate file labeled 'Manuscript'.

If you would like to make changes to your financial disclosure, please include your updated statement in your cover letter. Guidelines for resubmitting your figure files are available below the reviewer comments at the end of this letter.

If applicable, we recommend that you deposit your laboratory protocols in protocols.io to enhance the reproducibility of your results. Protocols.io assigns your protocol its own identifier (DOI) so that it can be cited independently in the future. For instructions see: https://journals.plos.org/plosone/s/submission-guidelines#loc-laboratory-protocols. Additionally, PLOS ONE offers an option for publishing peer-reviewed Lab Protocol articles, which describe protocols hosted on protocols.io. Read more information on sharing protocols at https://plos.org/protocols?utm_medium=editorial-email&utm_source=authorletters&utm_campaign=protocols.

We look forward to receiving your revised manuscript.

Kind regards,

Pasyodun Koralage Buddhika Mahesh

Academic Editor

PLOS One

**Journal Requirements:**

1. When submitting your revision, we need you to address these additional requirements.

Please ensure that your manuscript meets PLOS ONE's style requirements, including those for file naming. The PLOS ONE style templates can be found at

https://journals.plos.org/plosone/s/file?id=wjVg/PLOSOne_formatting_sample_main_body.pdf and

https://journals.plos.org/plosone/s/file?id=ba62/PLOSOne_formatting_sample_title_authors_affiliations.pdf

2. Please update your submission to use the PLOS LaTeX template. The template and more information on our requirements for LaTeX submissions can be found at http://journals.plos.org/plosone/s/latex.

3. We note that the grant information you provided in the ‘Funding Information’ and ‘Financial Disclosure’ sections do not match.

When you resubmit, please ensure that you provide the correct grant numbers for the awards you received for your study in the ‘Funding Information’ section.

4. Thank you for stating the following in the Acknowledgments Section of your manuscript:

Support for this project was provided by Vital Strategies’ Civil Registration and Vital Statistics and Data Impact programs through the Bloomberg Philanthropies Data for Health Initiative and Resolve to Save Lives. The authors thank the interviewees in the 13 countries for their time and insights; Victor Hugo Alvarez Castaño, Benjamin Clapham, Valens Nndagijimana Kagisha, Fatima Marinho, Raven Meade, Emmanuel Ngwakongnwi, Elizabeth Ortiz, and Moyeen Uddin, for assistance in study design and data collection and interpretation; Ellen Kuwana for assistance in drafting the manuscript; and Karen Schmidt for editorial support.

We note that you have provided funding information that is not currently declared in your Funding Statement. However, funding information should not appear in the Acknowledgments section or other areas of your manuscript. We will only publish funding information present in the Funding Statement section of the online submission form.

Please remove any funding-related text from the manuscript and let us know how you would like to update your Funding Statement. Currently, your Funding Statement reads as follows:

The author(s) received no specific funding for this work.

Please include your amended statements within your cover letter; we will change the online submission form on your behalf.

5. Thank you for uploading your study's underlying data set. Unfortunately, the repository you have noted in your Data Availability statement does not qualify as an acceptable data repository according to PLOS's standards.

At this time, please upload the minimal data set necessary to replicate your study's findings to a stable, public repository (such as figshare or Dryad) and provide us with the relevant URLs, DOIs, or accession numbers that may be used to access these data. For a list of recommended repositories and additional information on PLOS standards for data deposition, please see https://journals.plos.org/plosone/s/recommended-repositories.

6. Please amend either the title on the online submission form (via Edit Submission) or the title in the manuscript so that they are identical.

7. Your ethics statement should only appear in the Methods section of your manuscript. If your ethics statement is written in any section besides the Methods, please move it to the Methods section and delete it from any other section. Please ensure that your ethics statement is included in your manuscript, as the ethics statement entered into the online submission form will not be published alongside your manuscript.

8. If the reviewer comments include a recommendation to cite specific previously published works, please review and evaluate these publications to determine whether they are relevant and should be cited. There is no requirement to cite these works unless the editor has indicated otherwise.

[Note: HTML markup is below. Please do not edit.]

Reviewers' comments:

Reviewer's Responses to Questions

**Comments to the Author**

1. Is the manuscript technically sound, and do the data support the conclusions?

The manuscript must describe a technically sound piece of scientific research with data that supports the conclusions. Experiments must have been conducted rigorously, with appropriate controls, replication, and sample sizes. The conclusions must be drawn appropriately based on the data presented.

Reviewer #1: No

Reviewer #2: Yes

Reviewer #3: Yes

**********

2. Has the statistical analysis been performed appropriately and rigorously?

Reviewer #1: No

Reviewer #2: Yes

Reviewer #3: Yes

**********

3. Have the authors made all data underlying the findings in their manuscript fully available?

The PLOS Data policy requires authors to make all data underlying the findings described in their manuscript fully available without restriction, with rare exception (please refer to the Data Availability Statement in the manuscript PDF file). The data should be provided as part of the manuscript or its supporting information, or deposited to a public repository. For example, in addition to summary statistics, the data points behind means, medians and variance measures should be available. If there are restrictions on publicly sharing data—e.g. participant privacy or use of data from a third party—those must be specified.

Reviewer #1: Yes

Reviewer #2: Yes

Reviewer #3: Yes

**********

4. Is the manuscript presented in an intelligible fashion and written in standard English?

PLOS ONE does not copyedit accepted manuscripts, so the language in submitted articles must be clear, correct, and unambiguous. Any typographical or grammatical errors should be corrected at revision, so please note any specific errors here.

Reviewer #1: Yes

Reviewer #2: Yes

Reviewer #3: Yes

**********

5. Review Comments to the Author

Please use the space provided to explain your answers to the questions above. You may also include additional comments for the author, including concerns about dual publication, research ethics, or publication ethics. (Please upload your review as an attachment if it exceeds 20,000 characters)

**Reviewer #1:**  The article as a whole is more like a research report than an academic paper, and I am puzzled by the scientific nature of the methodology used in the article, and the relatively small amount of work that went into the article. Please enrich the article further by taking into account the comments of other reviewers, and I will give my next revision after the overall improvement.

**Reviewer #2:**  1. Correct typos/duplications: “low- and middle-income income countries”; “evidence-informed informed risk ratings.”

2. Harmonize spelling/usage: choose digitization or digitalization and use consistently; standardize “COVID-19” (no space before/after hyphen).

3. Add footnotes to define “Number of deaths recorded through 2022” (RMS-captured deaths vs. national totals; inclusion/exclusion of community deaths; duplication handling).

4. Figure Suggestion - Add a simple schematic/logic model showing inputs → processes → outputs → outcomes for RMS (with facilitators/barriers around it). This helps generalizability beyond COVID-19

5. “COVID -19” → “COVID-19” (remove stray spaces).

6. Country names are listed however quotes are region-attributed, add one sentence explaining the anonymization rationale, and to confirm transcripts were de-identified and stored securely with restricted access.

7. General Specificity Improvements may be prudent - namely with acronyms like first mention of CRVS, and throughout the table with footnotes

8. Check punctuation around ellipses in quotations and bracket conventions.

9. Replace passive/wordy phrases where meaning can be tightened (e.g., “were able to be operationalized” → “were operationalized”).

10. Ensure consistent use of Em-Dashes vs. Hyphens

**Reviewer #3:**  The manuscript provides an extremely valuable and technically sound qualitative process review of the deployment of Rapid Mortality Surveillance (RMS) in 13 different nations. The approach, which included double-blind coding and in-depth interviews, was carried out carefully and is suitable for this kind of study. The conclusions, which suitably draw from the thematic findings, offer compelling proof of the crucial roles that government ownership, digitalization, and interagency cooperation have in the success of RMS.

The question about statistical analysis is N/A because this is a qualitative study that focuses on implementation methods.

The manuscript's important findings are accessible since it is written in clear, standard English and presented in an understandable manner.

In terms of data access, the authors attest that Vital Strategies provides the interview transcripts that underpin the conclusions, exhibiting a dedication to data access while upholding essential confidentiality.

**********

6. PLOS authors have the option to publish the peer review history of their article (what does this mean?). If published, this will include your full peer review and any attached files.

If you choose “no”, your identity will remain anonymous but your review may still be made public.

**Do you want your identity to be public for this peer review?**  For information about this choice, including consent withdrawal, please see our Privacy Policy.

Reviewer #1: **Yes:**  Wentai Bi

Reviewer #2: **Yes:**  Shrihaan Chaudhary

Reviewer #3: No

**********

[NOTE: If reviewer comments were submitted as an attachment file, they will be attached to this email and accessible via the submission site. Please log into your account, locate the manuscript record, and check for the action link "View Attachments". If this link does not appear, there are no attachment files.]

To ensure your figures meet our technical requirements, please review our figure guidelines: https://journals.plos.org/plosone/s/figures

You may also use PLOS’s free figure tool, NAAS, to help you prepare publication quality figures: https://journals.plos.org/plosone/s/figures#loc-tools-for-figure-preparation.

NAAS will assess whether your figures meet our technical requirements by comparing each figure against our figure specifications.

# Author comment: Author response to Decision Letter 1

**DOI:** 10.1371/journal.pone.0333157.r002

19 Mar 2026

Reviewer #1: The article as a whole is more like a research report than an academic paper, and I am puzzled by the scientific nature of the methodology used in the article, and the relatively small amount of work that went into the article. Please enrich the article further by taking into account the comments of other reviewers, and I will give my next revision after the overall improvement.

Response: This was a both a summative evaluation and a utilization-focused process evaluation. It was a summative evaluation in that it looked back to systematically describe specific outcomes and test assumptions following Rapid Mortality Surveillance (RMS) implementation efforts during a global pandemic. It is utilization-focused process evaluation that assessed how RMS work was implemented and determined what components of RMS system design and implementation contributed to or hindered the production of timely, quality data that meets the goals of RMS. In that way, countries and global partners can use these results to inform future strategies and the refinement of global guidance on RMS.

As noted by Reviewer #3, the question about statistical analysis is N/A because this was a qualitative study with double-blind coding of in-depth interviews that focused on implementation methods.

Reviewer #2: 1. Correct typos/duplications: “low- and middle-income income countries”; “evidence-informed informed risk ratings.”

Response: This has been corrected.

2. Harmonize spelling/usage: choose digitization or digitalization and use consistently; standardize “COVID-19” (no space before/after hyphen).

Response: This has been corrected.

3. Add footnotes to define “Number of deaths recorded through 2022” (RMS-captured deaths vs. national totals; inclusion/exclusion of community deaths; duplication handling).

Response: This has been done.

4. Figure Suggestion - Add a simple schematic/logic model showing inputs → processes → outputs → outcomes for RMS (with facilitators/barriers around it). This helps generalizability beyond COVID-19

Response: This has been added to page 5

5. “COVID -19” → “COVID-19” (remove stray spaces).

Response: We have reviewed the document and confirmed there are no instances of stray spaces, “COVID-19” is consistently used.

6. Country names are listed however quotes are region-attributed, add one sentence explaining the anonymization rationale, and to confirm transcripts were de-identified and stored securely with restricted access.

Response: This has been done. The anonymization rationale is mentioned in a footnote to the first quote, and a sentence on data security has been added to the Methods section.

7. General Specificity Improvements may be prudent - namely with acronyms like first mention of CRVS, and throughout the table with footnotes

Response: We’ve ensured that each acronym is defined at first use. Additional details have also been added to Table headings, including footnotes where needed to define methodology or for added clarity.

8. Check punctuation around ellipses in quotations and bracket conventions.

Response: This has been done, we’ve ensured consistent spacing in each quotation.

9. Replace passive/wordy phrases where meaning can be tightened (e.g., “were able to be operationalized” → “were operationalized”).

Response: This point is well taken however in the example above the ability to be operationalized or not was an important distinction, as some countries tried to operationalize RMS systems but were unable. We’ve tightened the wording to “could” in this instance and reviewed for others.

10. Ensure consistent use of Em-Dashes vs. Hyphens

Response: This has been done.

Reviewer #3: The manuscript provides an extremely valuable and technically sound qualitative process review of the deployment of Rapid Mortality Surveillance (RMS) in 13 different nations. The approach, which included double-blind coding and in-depth interviews, was carried out carefully and is suitable for this kind of study. The conclusions, which suitably draw from the thematic findings, offer compelling proof of the crucial roles that government ownership, digitalization, and interagency cooperation have in the success of RMS.

The question about statistical analysis is N/A because this is a qualitative study that focuses on implementation methods.

The manuscript's important findings are accessible since it is written in clear, standard English and presented in an understandable manner.

Response: Thank you very much for the comprehensive and positive feedback, this is well noted.

Attachment Submitted filename: Response to Reviewers.docx

# Peer review: Decision Letter 1

**DOI:** 10.1371/journal.pone.0333157.r003

**Authors:** Pasyodun Koralage Buddhika Mahesh, Pasyodun Koralage Buddhika Mahesh

31 Mar 2026

A Multi-Country Qualitative Evaluation of Rapid Mortality Surveillance During the COVID -19 Pandemic

PONE-D-25-49179R1

Dear Dr. Congdon,

We’re pleased to inform you that your manuscript has been judged scientifically suitable for publication and will be formally accepted for publication once it meets all outstanding technical requirements.

Within one week, you’ll receive an e-mail detailing the required amendments. When these have been addressed, you’ll receive a formal acceptance letter and your manuscript will be scheduled for publication.

An invoice will be generated when your article is formally accepted. Please note, if your institution has a publishing partnership with PLOS and your article meets the relevant criteria, all or part of your publication costs will be covered. Please make sure your user information is up-to-date by logging into Editorial Manager at Editorial Manager® and clicking the ‘Update My Information' link at the top of the page. For questions related to billing, please contact billing support.

If your institution or institutions have a press office, please notify them about your upcoming paper to help maximize its impact. If they’ll be preparing press materials, please inform our press team as soon as possible -- no later than 48 hours after receiving the formal acceptance. Your manuscript will remain under strict press embargo until 2 pm Eastern Time on the date of publication. For more information, please contact onepress@plos.org.

Kind regards,

Pasyodun Koralage Buddhika Mahesh

Academic Editor

PLOS One

Additional Editor Comments (optional):

Reviewers' comments:

Reviewer's Responses to Questions

**Comments to the Author**

1. If the authors have adequately addressed your comments raised in a previous round of review and you feel that this manuscript is now acceptable for publication, you may indicate that here to bypass the “Comments to the Author” section, enter your conflict of interest statement in the “Confidential to Editor” section, and submit your "Accept" recommendation.

Reviewer #1: All comments have been addressed

Reviewer #2: All comments have been addressed

**********

2. Is the manuscript technically sound, and do the data support the conclusions?

The manuscript must describe a technically sound piece of scientific research with data that supports the conclusions. Experiments must have been conducted rigorously, with appropriate controls, replication, and sample sizes. The conclusions must be drawn appropriately based on the data presented.

Reviewer #1: Yes

Reviewer #2: Yes

**********

3. Has the statistical analysis been performed appropriately and rigorously?

Reviewer #1: N/A

Reviewer #2: Yes

**********

4. Have the authors made all data underlying the findings in their manuscript fully available?

The PLOS Data policy requires authors to make all data underlying the findings described in their manuscript fully available without restriction, with rare exception (please refer to the Data Availability Statement in the manuscript PDF file). The data should be provided as part of the manuscript or its supporting information, or deposited to a public repository. For example, in addition to summary statistics, the data points behind means, medians and variance measures should be available. If there are restrictions on publicly sharing data—e.g. participant privacy or use of data from a third party—those must be specified.

Reviewer #1: No

Reviewer #2: Yes

**********

5. Is the manuscript presented in an intelligible fashion and written in standard English?

PLOS ONE does not copyedit accepted manuscripts, so the language in submitted articles must be clear, correct, and unambiguous. Any typographical or grammatical errors should be corrected at revision, so please note any specific errors here.

Reviewer #1: Yes

Reviewer #2: Yes

**********

6. Review Comments to the Author

Please use the space provided to explain your answers to the questions above. You may also include additional comments for the author, including concerns about dual publication, research ethics, or publication ethics. (Please upload your review as an attachment if it exceeds 20,000 characters)

Reviewer #1: (No Response)

Reviewer #2: Excellent work. I appreciate the thoroughness and care that you have put in your revisions. All work has been done thoroughly and with great scientific rigor, and is technically sound.

**********

7. PLOS authors have the option to publish the peer review history of their article (what does this mean?). If published, this will include your full peer review and any attached files.

If you choose “no”, your identity will remain anonymous but your review may still be made public.

**Do you want your identity to be public for this peer review?**  For information about this choice, including consent withdrawal, please see our Privacy Policy.

Reviewer #1: **Yes:**  Wentai Bi

Reviewer #2: **Yes:**  Shrihaan Chaudhary

**********

# Editor report: Acceptance letter

**DOI:** 10.1371/journal.pone.0333157.r004

**Authors:** Pasyodun Koralage Buddhika Mahesh, Pasyodun Koralage Buddhika Mahesh

PONE-D-25-49179R1

PLOS One

Dear Dr. Congdon,

I'm pleased to inform you that your manuscript has been deemed suitable for publication in PLOS One. Congratulations! Your manuscript is now being handed over to our production team.

At this stage, our production department will prepare your paper for publication. This includes ensuring the following:

* All references, tables, and figures are properly cited

* All relevant supporting information is included in the manuscript submission,

* There are no issues that prevent the paper from being properly typeset

You will receive further instructions from the production team, including instructions on how to review your proof when it is ready. Please keep in mind that we are working through a large volume of accepted articles, so please give us a few days to review your paper and let you know the next and final steps.

Lastly, if your institution or institutions have a press office, please let them know about your upcoming paper now to help maximize its impact. If they'll be preparing press materials, please inform our press team within the next 48 hours. Your manuscript will remain under strict press embargo until 2 pm Eastern Time on the date of publication. For more information, please contact onepress@plos.org.

You will receive an invoice from PLOS for your publication fee after your manuscript has reached the completed accept phase. If you receive an email requesting payment before acceptance or for any other service, this may be a phishing scheme. Learn how to identify phishing emails and protect your accounts at https://explore.plos.org/phishing.

If we can help with anything else, please email us at customercare@plos.org.

Thank you for submitting your work to PLOS ONE and supporting open access.

Kind regards,

PLOS ONE Editorial Office Staff

on behalf of

Dr. Pasyodun Koralage Buddhika Mahesh

Academic Editor

PLOS One
